# Abundance and Distribution Patterns of *Thunnus albacares* in Isla del Coco National Park through Predictive Habitat Suitability Models

**DOI:** 10.1371/journal.pone.0168212

**Published:** 2016-12-14

**Authors:** Cristina Gonzáles-Andrés, Priscila F. M. Lopes, Jorge Cortés, José Luis Sánchez-Lizaso, Maria Grazia Pennino

**Affiliations:** 1 Department of Marine Sciences and Applied Biology, University of Alicante, Alicante, Spain; 2 Statistical Modeling Ecology Group (SMEG), Departament d'Estadística i Investigació Operativa, Universitat de València. Valencia, Spain; 3 Fisheries Ecology, Management and Economics Unit–FEME, Ecology Department, Federal University of Rio Grande do Norte, Natal, Brazil; 4 Centro de Investigación en Ciencias del Mar y Limnología (CIMAR), Universidad de Costa Rica, San Pedro, San José, Costa Rica; Hawaii Pacific University, UNITED STATES

## Abstract

Information on the distribution and habitat preferences of ecologically and commercially important species is essential for their management and protection. This is especially important as climate change, pollution, and overfishing change the structure and functioning of pelagic ecosystems. In this study, we used Bayesian hierarchical spatial-temporal models to map the Essential Fish Habitats of the Yellowfin tuna (*Thunnus albacares*) in the waters around Isla del Coco National Park, Pacific Costa Rica, based on independent underwater observations from 1993 to 2013. We assessed if observed changes in the distribution and abundance of this species are related with habitat characteristics, fishing intensity or more extreme climatic events, including the El Niño Southern Oscillation, and changes on the average sea surface temperature. Yellowfin tuna showed a decreasing abundance trend in the sampled period, whereas higher abundances were found in shallow and warmer waters, with high concentration of chlorophyll-a, and in surrounding seamounts. In addition, El Niño Southern Oscillation events did not seem to affect Yellowfin tuna distribution and abundance. Understanding the habitat preferences of this species, using approaches as the one developed here, may help design integrated programs for more efficient management of vulnerable species.

## Introduction

Pelagic ecosystems are undergoing extreme changes in their structure and functioning due to climate change, pollution and overfishing [1]. Fisheries, for example, now access and exploit remote areas, such as deep ocean habitats, as closer and more traditional fishing grounds get depleted [2].

Marine top predators, including marine mammals, sharks, large tuna and billfish, are declining worldwide at a rapid rate, which can largely be attributed to fisheries [3]. The loss of these taxa is expected to have important effects in pelagic ecosystems, influencing many other organisms throughout the food chain and their associated habitats [4]. While different management tools, such as Marine Protected Areas (MPAs), have been increasingly used to protect benthic species and habitats in coastal waters (e.g.: coral reefs) [5], the protection of pelagic ecosystems and top-predators has been widely overlooked, expect for a few examples [6]. This is mostly due to the intrinsic dynamics of these habitats and the high mobility of these species. MPAs specifically designed to protect the pelagic environment would be harder to enforce and systematically monitor, due to the remoteness of the majority of the pelagic ecosystems. Despite such difficulties, there are a few examples of MPAs that were established, intentionally or not, with the goal of protecting pelagic species.

Isla del Coco National Park, Costa Rica, is one of these examples. It is an uninhabited island, located 550 km southwest of the Pacific coast of Costa Rica, reached only after a 36h boat ride from the mainland. Isla del Coco was declared a national park in 1978 but the marine portion was only included in 1984. The park was declared a UNESCO World Heritage site in 1997, and the marine protected area was extended in 1991 and again in 2001. The park is also a Ramsar site since 1998. In 2011, a special management area was created around Isla del Coco National Park, the Seamounts Marine Management Area with a marine protected area of 9,640 km^2^ [7].

The island is a biodiversity hot-spot [8], due to a combination of features including climate, exposure to diverse ocean currents, and geology. The waters surrounding the island have a permanent and shallow thermocline, characterized by a high abundance of zooplankton and pelagic fish. Such features explain why Isla del Coco has the highest fish biomass in the tropics (7.8 tonnes/hectare), of which 85% are represented by apex predators [9].

Although Isla del Coco has been protected and monitored for over 20 years [10], illegal fishing of large pelagic species still occurs within the park’s limits [11]. Legal and illegal fisheries of these species are difficult to monitor all over Costa Rica’s Exclusive Economic Zone. A significant source of uncertainty follows from the fact that large foreign fishing fleets operate in the region [12], with foreign markets driving the demand [13]. Official data show that from 1990 to 2000s fishing fleets in Costa Rica have rapidly grown, with an increase in landings from around 18,000 to 34,500 t·year^-1^ [14]. The ratio of coastal (fishes and crustaceans) to pelagic (tunas and billfishes) landings changed from 3:2 to 1:4 [12]. Catches of large pelagic species have increased during the last decade, and currently they are about 50% of the reported landings.

Fishing fleets of Costa Rica catch five species of tuna, with the Yellowfin tuna (*Thunnus albacares*) making up the majority of the catch (84.97%) [15]. This large pelagic species [16, 17] is globally distributed over the tropical and subtropical oceans [18], and its distribution in the Eastern Tropical Pacific ranges from southern California USA, to Peru [19]. Yellowfin tuna have extremely large population sizes compared to other tunas and its migration occurs between the Atlantic and Indo- Pacific Oceans [19]. In addition, it is listed as "Near threatened" and "trend decreasing" by the IUCN Red List [20].

In this study, we explored the distribution and abundance of the Yellowfin tuna within the Isla del Coco MPA from 1993 to 2013, using visual census data. Specifically, we assessed if changes in the distribution and abundance of this species in the MPA are related with habitat characteristics, fishing intensity and climate, including El Niño-Southern Oscillation (ENSO) events and longer-term changes in the average sea surface temperature [21].

## Material and Methods

### *Yellowfin tuna* data

**Fig 1:**
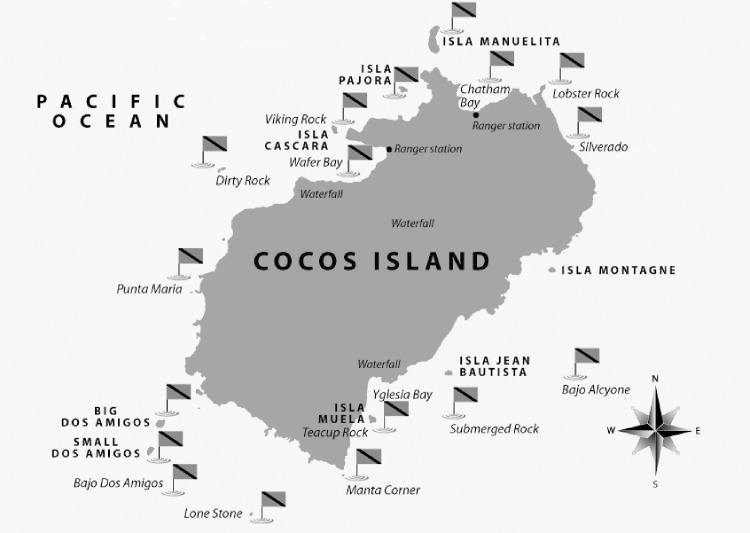
Map of the study area and the dive locations.

The Undersea Hunter Group [22] is a private diving company that operates in Isla del Coco and has one of the longest underwater visual censuses (UVC) for Yellowfin tuna, among others species, in the Eastern Tropical Pacific. Dives were performed between January 1993 and December 2013 at 17 different sites around Isla del Coco, resulting in 27,261 immersions (Fig 1 and Table 1).

**Table 1:** Summary of the number of dives for location and year around the island from the 1993 to 2013.

		1993	1994	1995	1996	1997	1998	1999	2000	2001	2002	2003	2004	2005	2006	2007	2008	2009	2010	2011	2012	2013	
**North**	I. Manuelita	103	90	124	138	75	167	345	344	256	523	410	298	531	519	608	543	549	624	901	723	630	
	Chatham B.	0	1	2	0	0	4	1	3	2	8	12	22	25	45	50	56	56	63	10	18	131	
	Lobster R.	17	17	18	20	15	54	56	36	31	72	69	24	53	62	59	46	84	58	117	136	137	
	Silverado	0	0	0	0	0	3	66	55	55	7	74	59	97	88	102	119	85	61	26	13	5	
** **	** **	**120**	**108**	**144**	**158**	**90**	**228**	**468**	**438**	**344**	**610**	**565**	**403**	**706**	**714**	**819**	**764**	**774**	**806**	**1054**	**890**	**903**	**11106**
**West**	I. Pajora	1	1	9	23	5	11	25	38	24	51	43	34	73	56	48	47	67	56	80	82	81	
	Viking Rock	20	17	9	13	8	19	43	38	30	89	63	62	58	64	99	50	45	40	91	77	71	
	Wafer B.	0	0	0	0	0	0	1	2	0	5	5	2	3	14	13	7	8	5	12	12	7	
	Dirty Rock	110	90	102	117	62	128	177	175	166	291	238	159	243	264	268	240	298	315	363	369	315	
	Maria P.	0	0	0	12	10	37	57	78	36	80	49	40	89	98	132	89	136	134	241	222	192	
** **		**131**	**108**	**120**	**165**	**85**	**195**	**303**	**331**	**256**	**516**	**398**	**297**	**466**	**496**	**560**	**433**	**554**	**550**	**787**	**762**	**666**	**8179**
**East**	Manta C.	12	4	1	5	0	10	7	3	3	15	10	2	8	2	4	6	5	2	8	1	1	
	Iglesias B.	0	0	0	0	1	3	0	2	1	1	4	1	0	0	1	4	1	0	0	8	0	
	Submerged R.	20	19	13	23	16	30	50	54	35	66	55	37	52	65	53	70	71	62	76	82	85	
	Alcyone	19	27	23	34	39	133	128	132	150	243	236	137	230	210	239	230	273	285	349	366	291	
** **		**51**	**50**	**37**	**62**	**56**	**176**	**185**	**191**	**189**	**325**	**305**	**177**	**290**	**277**	**297**	**310**	**350**	**349**	**433**	**457**	**377**	**4944**
**South**	Dos Amigos B	3	2	1	2	5	22	3	1	6	35	3	0	0	0	6	8	0	1	0	2	0	
	B. Dos Amigos	20	18	11	20	10	34	30	49	37	53	51	34	77	78	56	70	75	58	92	81	61	
	S. Dos Amigos	6	3	2	13	13	49	40	65	64	120	90	45	91	91	59	76	70	57	96	92	53	
	Lone Stone	25	17	20	24	21	61	57	64	39	91	41	22	56	29	17	25	45	12	27	22	7	
** **		**54**	**40**	**34**	**59**	**49**	**166**	**130**	**179**	**146**	**299**	**185**	**101**	**224**	**198**	**138**	**179**	**190**	**128**	**215**	**197**	**121**	**3032**
** **	Total	356	306	335	444	280	765	1086	1139	935	1750	1453	978	1686	1685	1814	1686	1868	1833	2489	2306	2067	

Each dive, always led by an experienced Divemaster during day light, averaged ~60 min and ranged in depth between 10–40 m. A total of 25 Divemaster led the dives along the time series. Although the dive protocol was not entirely standardized as in a scientific underwater visual census, the protocol was consistent throughout the period [21]. The maximum number of fish seen throughout the dive was recorded only when there were fewer than 100 individuals, whereas estimates were used otherwise (e.g., for schools of 1000 or more tunas).

Possible biases of false absences, which occur when an observer fails to record a present species, and recounting of individuals may have occurred during dives, however, such error would have been consistent throughout the survey period. In addition, as already demonstrated by White *et al*. (2015) [10], data collected by Divemasters can be a reliable way to discern trends in relative abundance, especially for large pelagic species that are easily identified [23].

Data were aggregated by year after excluding seasonality patterns with the Autocorrelation (ACF) and Partial Autocorrelation Function (PACF) in the R software [24].

In order to assess possible trends in catches, landing data of Yellowfin tuna were extracted for the time series 1993–2010. These data were available for all of Costa Rica’s Exclusive Economic Zone (EEZ) from the *Sea Around Us* website [25]. Landings are “reconstructed data” that combine official reports of the Food and Agriculture Organization of the United Nations (FAO) [14] and reconstructed estimates of Illegal, unreported and unregulated (*IUU*) fisheries data [26,27].

### Environmental data

Six environmental variables were considered as potential predictors of Yellowfin tuna abundance, including three climatic variables–Sea Surface Temperature (SST), sea surface salinity (SSS) and Chlorophyll-a concentration (Chl-*a*)–and three bathymetric features–depth, slope and distance to coast.

Bathymetric features were derived from the MARSPEC database [28]. MARSPEC is a world ocean dataset with a spatial resolution of 0.01 x 0.01 degrees developed for marine spatial ecology [29].

Depth and distance to coast are some of the main factors controlling species distribution and have been identified as predictors to determine spatial patterns of many species and in particular Yellowfin tuna [30, 31]. Slope is an index of seabed morphology and has been used as predictor of species distribution and of suitable habitats [32–35]. Low values of slope correspond to a flat ocean bottom (or areas of sediment deposition) while higher values indicate potential rocky ledges [33].

SST and Chl-*a* variables were extracted from different sensors as nightly monthly means and aggregated in yearly maps using the *Spatial Analysis* tool of ArcGIS 10 (Table 2).

**Table 2:** Predictor variables used fro modeling the abundance of the Yellowfin tuna in the Isla del Coco. SST = Sea Surface Temperature, SSS = Sea Surface Salinity, Chl-a = Chlorophyll-a.

Variable	Temporal resolution	Sensor	Platform
SST (°C)	1993–2006	AVHRR Pathfinder	http://www.neo.sci.gsfc.gov
SST (°C)	2007–2013	MODIS-Aqua	http://www.neo.sci.gsfc.gov
SSS	1993–2013	Standard Level Data: CTD (Surface)	World Ocean Database 2009
Chl-a (mg.m -3)	1993–1996	NEMO climatology model	http://www.nemo-ocean.eu/
Chl-a (mg.m -3)	1997–2013	SeaWiFS & MODIS-Aqua	http://oceancolor.gsfc.nasa.gov
Bathymetry (m)	-	SRTM30_Plus Bathymetry	http://www.marspec.org
Distance (km)	-	GSHHS Coastline	http://www.marspec.org
Slope (%)	-	Bathymetry	http://www.marspec.org

As no exhaustive and validated time series of SSS was available, the climatology of monthly SSS was downloaded from the World Ocean Database 2013 (WOA13) (Table 2).

Salinity and SST are strongly related to marine system productivity as they can affect nutrient availability, metabolic rates and water stratification [36]. Yearly maps of the SST can indicate temperature variations due to ENSO events, which happened in this area in 1997–1998, 2006–2007 and 2012 [37, 38].

Chl-*a* concentration was included in the analysis as an index of primary production of an ecosystem [39, 40]. Several studies have showed that primary production is an important factor that drives the Yellowfin tuna abundance and distribution [18, 41].

**Fig 2:**
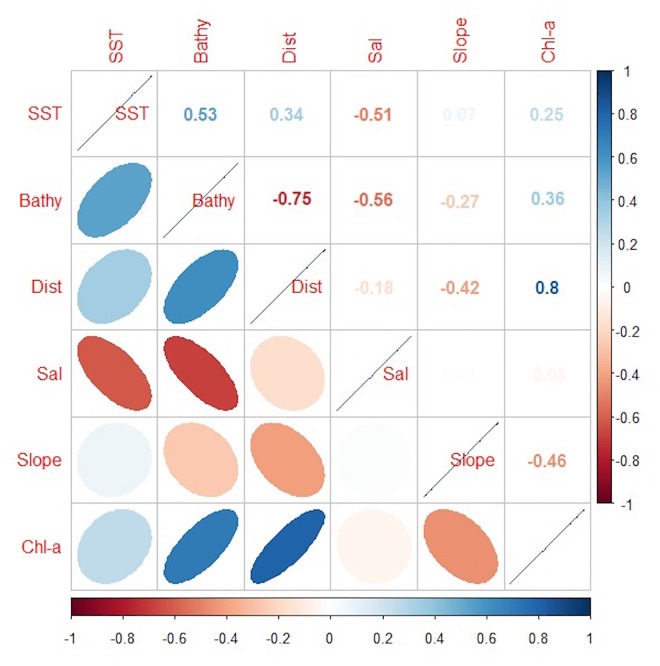
Cross-correlation matrix of the environmental variables included in the model.

All environmental variables were aggregated with a spatial resolution of 0.01 x 0.01 degrees. These variables were explored for collinearity, outliers, and missing data before their use in the models [42]. The variable distance to coast was highly correlated to depth (Pearson´s correlation, r > 0.75, p-value = 0.01) (Fig 2) and Chl-a (Pearson´s correlation, r > 0.8, p-value = 0.02), and thus, these variables were used alternatively in the models. Finally, to facilitate visualization and interpretation, the explanatory variables were standardized (difference from the mean divided by the corresponding standard deviation).

Finally, the Multivariate ENSO Index (MEI) was extracted from the NOAA website for the entire time series (2003–2013) (http://www.esrl.noaa.gov/). The Pearson and Spearman's correlations were computed between the MEI index and the Yellowfin tuna abundance in order to explore its effect on the species distribution. This index could not be included in the model because it does not have a spatial structure.

### Statistical models and model validation

We used hierarchical Bayesian hierarchical hurdle model to investigate how Yellowfin tuna spatial-temporal distribution and abundance respond to the explanatory variables. These types of models are implemented to deal with high numbers of zero in dives, in two stages: (i) modeling presence/absence in order to obtain the envelope of the predicted probability of presence of the species studied and (ii) modeling the number of individuals (*i*.*e*., count data) of the studied species only in areas where species were predicted to be present [43]. The first stage was modeled using a binomial distribution and the second with a Poisson distribution.

For both stages, the candidate explanatory variables included all environmental variables, the unstructured random effect of the year, a spatially structured random effect, an observer random effect and all possible interaction terms. The observer random effect is included in the model to account for a possible non-independence in the observations that could explain the remaining potential source of variation in the number of Yellowfin tuna sighted, due to the observers themselves (e.g.: personal experience) or due to unobserved survey characteristics (e.g.: water visibility). Finally, in order to account for the sampling effort variability among dive locations and year an offset was included in the second stage of the model.

A vague zero-mean Gaussian prior distribution with a variance of 100 was used for all of the parameters involved in the fixed effects, while for the spatial effect a zero-mean prior Gaussian distribution with a Matérn covariance structure was assumed (see Muñoz et al. 2013 [44] for more detailed information about spatial effects).

For each particular parameter, a posterior distribution was obtained. Unlike the mean and confidence interval produced by classical analyses, this type of distribution enables explicit probability statements about the parameter. Thus, the region bounded by the 0.025 and 0.975 quantiles of the posterior distribution has an intuitive interpretation: for a specific model, the unknown parameter is 95% likely to fall within this range of values.

Once the inference has been carried out, we predicted the species abundance in the rest of the area of interest for the entire year using Bayesian kriging, which allows for the incorporation of parameter uncertainty into the prediction process by treating the parameters as random variables (see Muñoz et al. 2013 [44] for more detailed information about this approach).

Variable selection was performed beginning with all possible interaction terms, but only the best combination of variables was chosen. Such choice was based on two criteria: Deviance Information Criterion (DIC) [45] and on the cross validated logarithmic score (LCPO) measure [46]. Specifically, DIC was used as a measure for goodness-of-fit, while LCPO as a measure of the predictive quality of the models. DIC and LCPO are inversely related to the compromise between fit, parsimony and predictive quality.

All the analyses were performed using the Integrated Nested Laplace Approximation (INLA) methodology [47] and INLA package [48], in R software [24].

We used two separated approaches to assess the predictive accuracy of the selected model. Firstly, the predicted and observed values using the full dataset were compared. Secondly, a 10-fold cross validation using a random half of the dataset was performed to build the model and the remaining data to test the prediction [49].

Two statistics were calculated for both approaches: Pearson’s correlation coefficient *r* and the average error (AVEerror). Pearson’s correlation coefficient, *r*, measures the linear dependence between predicted and observed values. It can vary from -1 to 1, with 1 representing a perfect positive correlation between the two datasets. The AVEerror represents the mean error between observed and predicted values. The closer this statistic is to zero, the better the prediction [50].

## Results

### Bayesian models

Yellowfin tuna abundance was mainly explained by bathymetry, Chl-*a*, SST, slope, the interaction between SST and Chl-*a*, and the random spatial and temporal effects (Table 3), according to the model with the best fit (based on the lower DIC and LCPO). Distance from the coast and salinity were not relevant variables, as all models with these effects showed higher DIC and LCPO than those without them.

**Table 3:** Numerical summary of the posterior distribution of the fixed effects for the best model of the Yellowfin tuna.

Predictor	Mean	SD	Q_0.025_	Q_0.5_	Q_0.975_
Intercept	1.28	0.45	0.23	1.12	2.13
Bathymetry	-1.10	0.33	- 2.34	-0.98	-0.11
Slope	0.87	0.13	0.11	0.81	1.45
SST	0.84	0.27	0.08	0.77	1.14
Chl-a	1.42	0.22	0.33	1.40	2.54
Chl-a x SST	1.94	0.15	0.13	0.89	2.56

This summary contains mean, the standard deviation (SD), the median (Q_0.5_) and a 95% credible interval (Q_0.025_—Q_0.975_), which is a central interval containing 95% of the probability under the posterior distribution. Chl-a = Chlorophyll-a concentration, SST = Sea Surface Temperature.

Yellowfin tuna showed to be more abundant in shallower waters (posterior mean = -1.10; 95% CI = [-2.34, -0.11]), according to the model. Also, higher abundance of Yellowfin tuna should be expected in warmer waters (posterior mean = 0.84; 95% CI = [0.08, 1.14]), with higher primary productivity (i.e., higher concentrations of Chl-a) (posterior mean = 1.42; 95% CI = [0.33, 2.54]) and more complex bottoms (e.g. rocky ledges). The interaction between SST and Chl-a concentration showed a positive relationship (posterior mean = 1.94; 95% CI = [0.13, 2.56]): Yellowfin tuna abundance increased in warmer waters with higher concentration of Chl-a.

**Fig 3:**
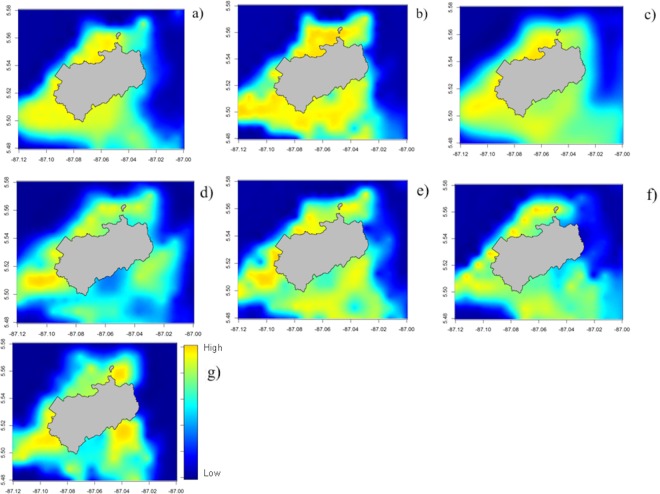
**Predictive maps of the abundance of the Yellowfin tuna (*Thunnus albacares*) aggregated in intervals of 3 years: (a) 1993–1995; (b) 1996–1998; (c) 1999–2001; (d) 2002–2004; (e) 2005–2007; (f) 2008–2010; (g) 2011–2013**.

Maps of the predicted abundance of Yellowfin tuna in sampled and non-sampled areas were generated for intervals of 3 years (1993–1995; 1996–1998; 1999–2001; 2002–2004; 2005–2007; 2008–2010; 2011–2013). The spatial patterns of Yellowfin tuna abundance are consistent with the model predictions, as higher abundances were predicted in shallower waters, closer to the coast where the productivity is higher and where the seabed shows some structuring (Fig 3). Predictive maps suggest a decreasing trend in the abundance of Yellowfin tuna between 1993 and 2013, but such trend showed no correlation with the ENSO events (Fig 3).

In addition, Pearson and Spearman's correlations (Table 4) confirmed that there was no influence of the ENSO events of the Yellowfin Tuna abundance. Indeed, both the SOI and MEI indexes were not correlated with the Yellowfin tuna abundance.

**Table 4:** Correlation between MEI and SOI indexes and the Yellowfin tuna abundance from 1993 to 2013.

	Spearman’s correlation	Pearson’s correlation
MEI Index	r = -0.02, p-value = 0.01	r = 0.06, p-value = 0.01
SOI Index	r = -0.04, p-value = 0.02	r = 0.05, p-value = 0.02

The selected model presented a good fit, showed by the high values for the Pearson’s correlation coefficient both for the original dataset (0.71, p-value = 0.01) and for the cross validation done with half of the dataset (0.77, p-value = 0.01). Likewise, low values for the AVEerror were achieved in both the original (AVEerror = 0.03) and in the cross validation (AVEerror = 0.02) datasets.

### Landing data

The temporal trend of the landings of the Yellowfin tuna for the entire Costa Rica EEZ shows a clear increasing in the catches of this species from 1999 onward, followed by a stabilization at lower levels in the last years of the times series, particularly after 2007 (Table 5). On the other hand, the visual census data for Isla del Coco suggest that the number of individual Yellowfin tunas was higher in the first years of observation, reached a peak in 1997, and then decreased to its lowest level in 1998, remained at this level since then (Table 5).

**Table 5:** Temporal trends of landings and sightings of Yellowfin tuna. Landings (in tonnes) refer to the entire Costa Rica EEZ and refer to the period 1993 to 2010.

Year	Landing data	Tuna Abundance
1993	72.458.000	1300
1994	78.090.000	16450
1995	74.310.000	1670
1996	75.428.000	1918
1997	64.308.000	6400
1998	65.210.000	640
1999	68.995.000	310
2000	103.669.000	280
2001	80.475.000	970
2002	114.745.000	550
2003	109.885.000	1300
2004	87.978.000	1020
2005	91.072.000	2610
2006	86.791.000	2500
2007	64.546.000	6380
2008	67.052.000	940
2009	65.809.000	680
2010	65.458.000	2200

## Discussion

Underwater survey censuses of the Yellowfin tuna (*Thunnus albacares*) performed along 21 years were used to improve our understanding of habitat selection by this species and its changes in distribution and abundance over time in Isla del Coco National Park. These data represent the only long-term sighting data for Yellowfin tuna, not only for Isla del Coco, but for the entire Eastern Tropical Pacific. The analyses carried out (hierarchical Bayesian approach) represent the state-of-the-art to predict species abundance, while they also account for a spatial temporal component, an important effect commonly overlooked in most studies.

The strongest predictors of the Yellowfin tuna habitats in Isla del Coco were chlorophyll and water temperature. These two factors are strongly related with ecosystems primary production, by influencing the availability of food [36, 39, 40]. This result is consistent with other studies that had already suggested that Yellowfin tuna is highly influenced by the primary production [39,51,52]. Another important factor that affects the distribution of this species is the seabed topography and structure. Isla del Coco sits atop the Coco Volcanic Cordillera, a submarine mountain offshore the southern part of Costa Rica [53,54], which apparent attracts aggregation of Yellowfin tuna [55–57]. Indeed, seamounts may act as midocean reference points that occasionally harbor increased prey densities that attract this species [58, 59].

Previous studies have observed the preference of Yellowfin tuna for shallower waters [60,61], which was confirmed here, as all predictive maps estimated higher abundances in depths between 20–80 m, and lower abundances between 90–100 m. Such findings are also in line with previous tagging studies that showed that this fish spent 85% of its time in waters close to the thermocline [61] in Isla del Coco, which happens around 50 m deep [62,63].

The predictive maps also showed that the southeast part of the island holds higher abundance of Yellowfin tuna. Since slope and bathymetry vary little around Isla del Coco [64] the preference for these areas could be due to a higher average concentration of nutrients. The south side of the island is influenced by the North Equatorial Counter Current [65, 66] and high values have been reported from that area [67], which could generate a higher productivity in the southeast.

Whereas Yellowfin tuna distribution is affected by the water temperature, probably due to its effect on productivity, it does not seem to be affected by the ENSO events. Only in the second group of years (1996–1998) there is a higher abundance that could be due to the 1997 ENSO event, as already demonstrated by Torres-Orozco et al. (2006) [68] in the Gulf of California. This could be because the study area is probably in the middle of the distribution range of this species, where climate changes do not significantly affect its distribution. Further studies with data sampled in a larger area should be done, to better understand the effects of ENSO on the entire distribution of Yellowfin tuna.

The temporal and spatial trends found in this study clearly indicate a decreasing pattern in the abundance of this species and shifts in its geographical distribution. This decrease could not be due to a possible "learning effect" of the observers. Although divers acquire more experience with time and learn to identify and count individuals better, the Bayesian analysis did not select the observer effect as possible predictor in the final model, suggesting that eventual variability in the data due to divers is low.

Moreover, the increasing trend of landings of this species in the 2000s in all the Costa Rica EEZ could be the direct cause of the lower sightings of this species in the Island. Isla del Coco is recognized as an example of a successful MPA and a well-known site for worldwide divers for large pelagic watching [5, 10, 69]. This fact could imply, as already suggested by White et al. (2015) [10], a problem of shifting baselines, with recreational divers failing to recognize how much of the megafauna of Isla del Coco has already been lost.

It is unclear if the current decreasing trend of the Yellowfin tuna in Isla del Coco is an indicative of an ineffective management of the MPA and/or an inducted effect of the fisheries that operate in the entire Costa Rica EEZ. Indeed, although management efforts have increased in the past decade, illegal fishing still occurs within the island’s waters [11, 70]. However, has this species has a wide distribution species, animals that get to Coco crossed waters fished by many other countries. The decrease could also be due to fishing anywhere else on the route to Coco.

On the other hand, hot-spots of Yellowfin tuna in Isla del Coco could also be an indication of a positive effect of the MPA that has preserved this species in the waters surrounding the island [70]. A significant increase in the abundance of this species will likely be achieved only through much larger and strategic protected areas that also consider the life cycle, as this is a highly mobile pelagic species subjected to intense fishing mortality.

Further studies are needed to extend the spatial scale of the predicted distribution of this high mobility species and to understand if the possible fishing effects are directly connected with the decreasing abundance of this species. However, understanding the habitat preferences of this species using approaches as the one developed here may help design integrated programs for more efficient management of marine resources.
